# Reproductive Performance in a Selected Sample of Dairy Farms in Una-Sana Canton, Bosnia and Herzegovina

**DOI:** 10.1155/2020/2190494

**Published:** 2020-03-16

**Authors:** Adis Softic, Adam Dunstan Martin, Eystein Skjerve, Nihad Fejzic, Teufik Goletic, Aida Kustura, Erik Georg Granquist

**Affiliations:** ^1^Department of Food Safety and Infection Biology, Faculty of Veterinary Medicine, Norwegian University of Life Sciences, P.O. Box 8146, 0033 Oslo, Norway; ^2^Department of Zootechnics and Poultry Diseases, Faculty of Veterinary Medicine, University in Sarajevo, Zmaja od Bosne 90, 71000 Sarajevo, Bosnia and Herzegovina; ^3^Department of Production Animal Clinical Sciences, Faculty of Veterinary Medicine, Norwegian University of Life Sciences, P.O. Box 8146, 0033 Oslo, Norway; ^4^Department of Epizootiology, Faculty of Veterinary Medicine, University in Sarajevo, Zmaja od Bosne 90, 71000 Sarajevo, Bosnia and Herzegovina

## Abstract

**Background:**

The production of milk and dairy products and their placement on the market represent a constant profit for the farmers/producers in Bosnia and Herzegovina (BH). The profitable operation of the dairy farms is influenced by the reproductive performance of the lactating animals. This study assessed individual animal reproductive characteristics in selected dairy farms and described their reproductive performance indicators.

**Results:**

The median age at first insemination was 493 days (5^th^–95^th^ percentile range 429–840), while the age at first calving was 802 days (5^th^–95^th^ percentile range 708–1168). The median pregnancy proportion at first insemination was 40% (5^th^–95^th^ percentile range 17–62), while the cumulative pregnancy rate calculated at day-60, day-80, day-100, and day-120 showed that approximately 64% of all pregnancies happened before day-120. The calculated interservice intervals showed that approximately 69% of the repeat breeding animals came back to the oestrus in the period of 18 to 24 days. This is an indication of very good oestrus detection in selected dairy farms. The mean number of services per pregnancy was 2.61 (range 1–12). The median calving-to-first-insemination interval was 62.5 days (5^th^–95^th^ percentile range 16–408). The calving-to-conception interval was 101 day (5^th^–95^th^ percentile range 36–506). Finally, the calving interval was 385 days (5^th^–95^th^ percentile range 329–773).

**Conclusions:**

There is a need for an organised, regular, and more comprehensive recording system for the reproduction of dairy cattle among dairy farms in Una-Sana Canton. The calculated reproductive measures indicated an undulant trend in reproductive performance among selected dairy farms in Una-Sana Canton. Knowing the apparent reproductive indicators described in this study, the farmers and veterinary authorities may identify and correct areas in their management that contribute to the reproductive underperformance.

## 1. Introduction

The reproductive performance of modern dairy cow worldwide has decreased over the past 50 years. The observed reproductive decline has been partially explained by the intensification of the production, with continuously higher milk yield and larger herds [1]. One recent study suggested that the declining trend in reproductive performance has slowed down [2], whilst others have suggested that the correlation between increased production and decreased reproductive performance is still significant although the effect is small and modulated at the herd level [3]. Milk quotas within the European Union (EU) were lifted in 2015 [4]. This has led to an expansion in milk production in Central and Western Europe. Increased milk production represents another stress which can eventually lead to the decline in reproductive performance in dairy cattle in high-producing regions of EU. This potential loss can be overcome by the improvement of key areas in dairy cow fertility management such as genetic selection, nutritional management, control of infectious diseases, and control of cow and bull fertility [5]. The expanding milk production in South-Eastern Europe and Baltic countries may reduce some pressure on already high-producing regions of the EU, but the dairy industry may also be faced with challenges in preventing the potential reproductive losses.

Although not a member of the EU, BH plays a role in the EU milk supply, having received the official permit for the export of milk and dairy products to the EU [6]. Consequently, the intensification of the production, with continuously higher milk yield and potential consolidation of dairy herds are primary aspirations and future plans of the dairy industry in BH. Dairy production, in general, has been gradually increased in BH over the last 20 years, although there were considerable annual variations in the amount of produced milk and the number of dairy cows [7]. There was a continual decrease in the number of registered dairy cows in BH throughout the period 2006–2016, while the total milk production increased, reaching 701 million litres in 2016 [8].

However, a number of limiting factors still prevent the country from maximising its potential milk production. The agricultural land and farm properties are fragmented with extensive and unwieldy farming practices, and stocking densities are typically low [9]. Also, BH is still a country in political and economic transition, and agricultural conditions for more intensive production growth are yet not directly comparable with EU countries (EUROSTAT, 2016). These limiting factors are partially mitigated by the national programmes that are results of the alignment of country legislation with the EU regulations. The optimal bovine reproduction remains one of the essential factors required to achieve the goals of the dairy industry in BH. Hence, there is a need for the monitoring of reproductive performance indicators in order to maximise the production efficiency and milk yield, but also to comprehend the effect size of changes caused by the intensification in the production. However, the information on the current status of reproductive performance is largely unknown in dairy herds in BH.

Traditionally, dairy farmers aim to produce one calf per cow per year to ensure dairy herd replacement and to optimise milk production. However, herd health programmes (including breeding programmes, nutritional strategies, and biosecurity), data management strategies, precision farming systems, and databases are scarce or absent in most farms in BH. In addition, different legislation levels (state, entity, or cantonal) and poorly developed herd health services, breeding organisations, and automatic recording systems seem to additionally hamper the dairy industry development in BH. Establishing fertility and merit indices are important for monitoring individual, herd and population reproduction efficacy. Moreover, these indices partially contribute to a better understanding of the causality of reproductive problems and also assist decision-making process and economic evaluation in the dairy production [10, 11]. Internationally, low pregnancy rates have been accompanied by a reduction in bulk milk production and calves born per year, which negatively influences the economic profitability of the dairy farm [12]. Since the reproductive performance of dairy cattle may be influenced by a number of individual and environmental factors, substantial variations are seen across the country.

Therefore, this study aimed at identifying and describing the individual animal reproductive performance in a subset [13] of investigated dairy herds in Una-Sana Canton of BH.

## 2. Materials and Methods

### 2.1. Study Design and Study Population

**Figure 1:**
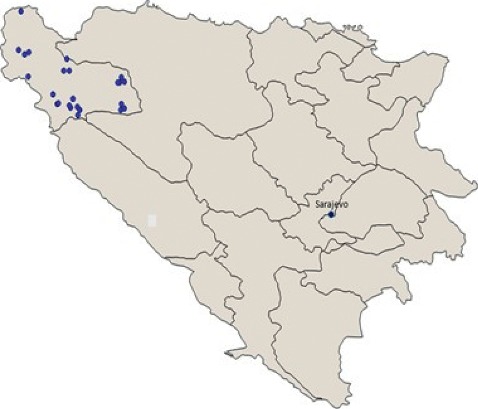
Distribution of selected dairy farms in Una-Sana Canton, BH.

**Figure 2:**
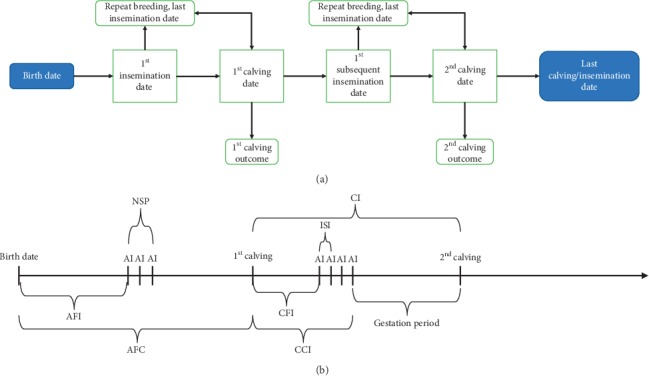
(a) Scheme of the recording of reproductive events in selected farms in Una-Sana Canton; (b) the calculation of reproductive indicators from available farm records. AI, artificial insemination; AFI, age at first insemination; AFC, age at first calving; NSP, number of services per pregnancy; ISI, interservice intervals; CFI, calving to first insemination interval; CCI, calving to conception interval; CI, calving interval.

The follow-up study was carried out in the north-western part of BH (Una-Sana Canton) (Figure 1). Farm visits and data collection were completed in the period November–December 2016. To describe individual animal reproductive performance, individual timeline data were recorded. The timeline data referred to the list of all reproductive events in chronological order from birth to either the end of the cow's reproductive life or the last reproductive event. Reproductive events were recorded as calendar dates, and the selection strategy is schematically presented in Figure 2.

The target population comprised of commercially farmed dairy cows in Una-Sana Canton. Since there was no complete list of all dairy farms in Una-Sana Canton, the sampling frame consisted of dairy farms from the available municipal lists of cooperatives with dairies. In addition, the selection of farms was based on a subset of dairy farms assembled retrospectively from a previous study on farm management and reproductive infections in dairy cattle, as described in [13]. The general inclusion criterion was the existence of an on-farm written recording system for reproductive data at the individual animal level. The study sample consisted of animals that had entered their reproductive life and were enrolled in the farm records. Visited farms with only memorised data or insufficient written data were excluded from the study. Newborn animals, noninseminated heifers, and animals with incomplete or missing timeline data were not included in the study. The aim was to retrospectively collect all individual data for five years. Given all farms that met the inclusion criteria had appropriate written data in the period 2009–2016, this period was defined as the study period of interest. Animals whose timeline did not start with the birth date, but whose available data had no interruption of the chronological continuity were kept in the study. In such cases, the chosen starting point on the timeline was either (i) the date of the first service that resulted in a pregnancy and calving or (ii) the calving date, after which they started a new reproductive cycle or finished their reproductive life. Newly introduced animals were included in the study if their records started with the date of introduction in the farm and continued with the reproductive events.

### 2.2. Data Collection and Calculation

A data collection form developed before the study was initiated for recording reproductive events. Farms' handwritten papers, diaries, and daily tables were used in the preparation of the database. Each animal timeline data were reviewed and put into the database only if it met the inclusion criteria. The database was established in a Microsoft Excel® spreadsheet. After calculating and reviewing data in Excel using filter functions and pivot analyses, data were transferred to Stata SE/15 for Windows (StataCorp, College Station, TX) for further analyses. Reproductive measures calculated in this study are age at first service, age at first calving, pregnancy proportion at first service, number of services per pregnancy, calving to first service interval, calving to conception interval, and calving interval. A heifer was considered as a sexually mature animal with no previous calvings. The age at first service is relevant only for heifers and was calculated from the following formula:

(1) $$\begin{array}{c} \text{age}\left(1\text{st service}\right)=\text{first service date}-\text{birth date.} \end{array}$$

Subsequently, age at first calving was calculated as

(2) $$\begin{array}{c} \text{age}\left(1\text{st Calv}\right)=\text{first calving date}-\text{birth date}. \end{array}$$

These measures were calculated and presented at the farm level. The pregnancy proportion (PP) at the first service was calculated from the available data. This proportion was calculated for all included farms in accordance with the following formula:

(3) $$\begin{array}{c} \text{PP}=\frac{\text{number of pregnant heifers}}{\text{number of inseminated heifers}}\times 100. \end{array}$$

A cow was considered pregnant if she did not return to oestrus after breeding and before calving or if the pregnancy was confirmed by rectal palpation. Cows that were inseminated more than three times at regular intervals were classified as repeat breeder cows [14]. Since natural service was uncommon but was appropriately recorded, natural breeding and artificial insemination were compiled as one measure. In repeat breeding cows, all artificial insemination or natural service events were coded as 0 or 1 (cow bred no/yes). The sum of all insemination events followed by the calving date was the number of inseminations per pregnancy at the individual animal level. The average annual number of inseminations per pregnancy was calculated at the farm level as follows, where *n* represents the number of cows at the farm at a given time (year).

(4) $$\begin{array}{c} \text{Average no. of services per pregnancy}=\frac{1}{n}\sum \left(\text{services per pregnancy}\right).\; \end{array}$$

Further, the insemination index was calculated for all farms, as an overall average number of services per pregnancy (artificial insemination or natural breeding) of all cows present at the farm (data not shown). Multiple serviced animals without subsequent calving and animals not confirmed to be pregnant were excluded from the calculation. Additionally, the interinsemination interval was defined as the number of days between two consecutive inseminations/services. It was calculated in repeat breeding animals to identify the characteristics of oestrus and oestrus detection.

The calving-to-first-service interval (CFI) was defined as the number of days from the calving until the cow's first artificial insemination or natural service:

(5) $$\begin{array}{c} \text{CFI}=\text{first service after calving}-\text{calving date}. \end{array}$$

This reproductive measure was calculated at the individual and farm level. Also, the CFI is presented as an annual average at the farm level. Similarly, the calving-to-conception interval (CCI) was the number of days from calving until the effective insemination service.

(6) $$\begin{array}{c} \text{CCI}=\text{effective insemination service}-\text{calving date.} \end{array}$$

This reproductive measure was calculated and presented at the individual and farm level. For the multiparous animals, the calving interval (CI) was calculated as follows:

(7) $$\begin{array}{c} \text{CI}=\text{calving date}\left(n+1\right)-\text{calving date}\left(n\right). \end{array}$$

Given the previously calculated measures, we were able to check the gestation period of all individuals that had two consecutive calvings. The gestation period was defined as the difference between CI and CCI. Cows with the gestation period below 270 days were considered as cows that experienced foetal loss. Such cows were kept in the calculation only if they had subsequent calving after the foetal loss. Consequently, their CI was considered as the difference between two normal calvings. The CI was then averaged and presented as the average annual CI in accordance with the formula:

(8) $$\begin{array}{c} \text{CI}\left(\text{average}\right)=\frac{1}{n}\;\sum \left(\text{CI}n\right), \end{array}$$

where *n* represents the number of cows in the herd at a given time (year). Additionally, the calving index was calculated as an overall average CI of all cows in the farm. The moderate continental climate in BH is characterised by four annual seasons: spring (March 21^st^–June 21^st^), summer (June 21^st^–September 23^rd^), autumn (September 23^rd^–December 21^st^), and winter (December 21^st^–March 21^st^). Accordingly, we determined the calving season for each calving and recorded it in the dataset afterwards.

### 2.3. Descriptive Statistics

The median and 5–95 percentile range were calculated for continuous variables. Frequencies were calculated for dichotomous and categorical variables. The Kruskal–Wallis test was used for assessing the statistical difference between continuous dependent (reproductive measures) and categorical independent (farms) variables. Also, data were presented using box plots, bar graphs, and histograms.

## 3. Results

Twenty-four managers of dairy farms were approached, and twenty-two of them (92%) agreed to participate in this study. Five of twenty-two dairy farms (23%) were excluded due to unreliable data recordings, leaving 17 farms in the final dataset. For individual cows, 57% (310/544) of individual data recordings were excluded from the study due to inappropriate written data or only providing memorised data. Finally, recordings for 234 animals from 17 dairy farms were confirmed eligible and included in the study. The median number of animals per farm was 20 (range 9–40). Further, the median number of heifers per farm was five (range 0–12), while the median number of cows per farm was 14.5 (range 4–26). The most frequent breeding method was artificial insemination, and it was represented in all of the included farms. The use of natural service sires was the additional option in case of long-lasting failures in conception. All farmers (17/17) reported the use of natural service sires at least for one cow in the period 2009–2016. The most common breed was Simmental (126/234) and Simmental crossbreeds (77/234), while the rest of the animals were Holstein-Friesian and Holstein-Friesian crossbreeds (26/234).

**Figure 3:**
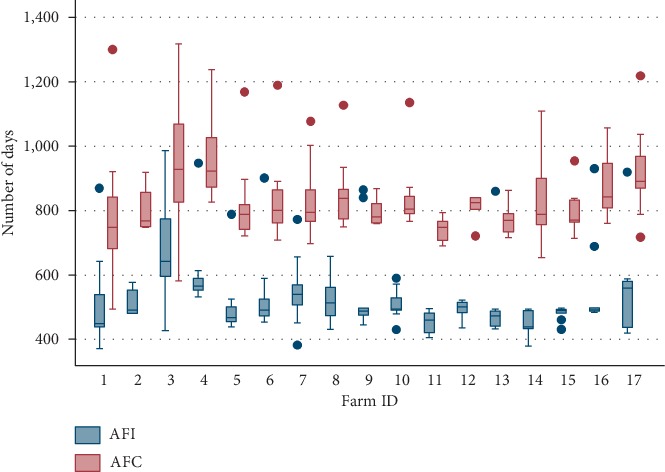
Distribution of the age-at-first-insemination (artificial or natural breeding) and the age-at-first-calving in heifers among selected dairy farms in Una-Sana Canton, BH. Some investigated farms had heifers with AFI and AFC far removed from the mass of farm data, indicating certain reproductive problems. Those heifers are referred to farm outliers (dots in the graph).

**Figure 4:**
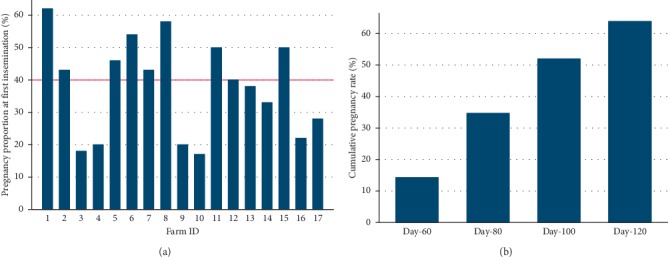
The pregnancy proportion expressed as (a) the pregnancy proportion for heifers at the first insemination service and (b) the population-level cumulative pregnancy rate at day-60, day-80, day-100, and day-120 after the previous calving in the selected dairy farms in Una-Sana Canton, BH.

**Figure 5:**
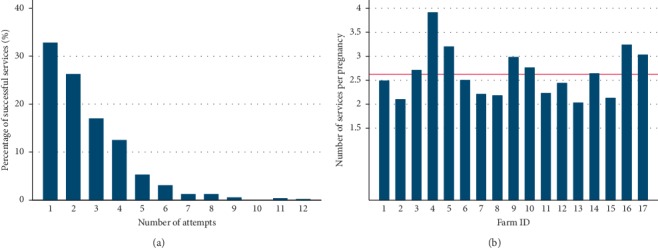
The number of services (artificial insemination and natural breeding) per pregnancy expressed as (a) the percentage of successful services distributed by the number of attempts and (b) the mean number of services per pregnancy distributed by the dairy farm in the selected area of Una-Sana Canton, BH.

**Figure 6:**
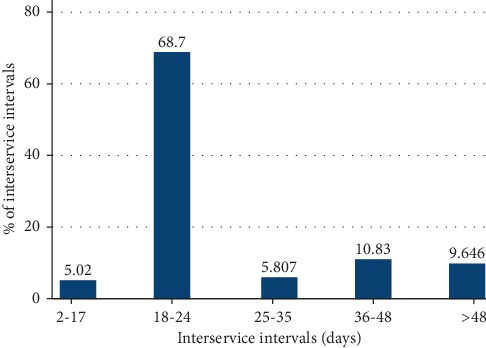
Interservice intervals histogram. Repeat breeding services recorded and compiled at the population level showed that 68.7% of returns to the oestrus occurred in 18 to 24 days period. Considering good oestrus detection, the target is set on 55% as described in [15].

The median AFI was 493 days (5^th^–95^th^ percentile range 429–840), and the median AFC was 802 days (708–1168).  Figure 3 shows the variations in the AFI and the AFC among selected dairy farms. The median pregnancy proportion at the first insemination service was 40% (17–62) (Figure 4(a)). The cumulative pregnancy rates after day-60, day-80, day-100 and day-120 are shown in Figure 4(b). The cumulative pregnancy rate after day-120 was approximately 64% at the population level. A total of 33% of the first inseminations (artificial and natural) resulted in pregnancy (Figure 5(a)), while the mean number of services per pregnancy (NSP) varied substantially over selected dairy farms (Figure 5(b)). A total of 68.7% of calculated interservice intervals were distributed in the range of 18 to 24 days, while 10.8% of them were in the range of 36 to 48 days (Figure 6). The CFI and the CCI were unevenly distributed within and between selected farms (Table 1). The median CI and its distribution over selected farms are shown in Table 1. The median CFI at the population level was 62.5 days (31–408), while the median CCI was 101 days (36–506). Finally, the median CI for all selected farms was 385 days (range 329–773 days) (Table 2.) Since we were able to track reproductive events in the period 2009–2016, the annual and overall distributions of CFI, CCI, and CI are shown in Table 2. All visited farmers followed all-year-round calving, with an approximately equal proportion of calving per each season. We have compiled all dates of recorded calvings, and found out that 19% of them (108/573) have occurred in spring (March 21^st^–June 21^st^), 29% (163/573) in summer (June 21^st^–September 23^rd^), 26% (149/573) in autumn (September 23^rd^–December 21^st^), and 27% (153/573) in winter (December 21^st^–March 21^st^).

## 4. Discussion

Dairy farming in BH faces several challenges in reproductive management of dairy cattle to maintain milk production and farm profitability. The importance of reproductive performance benchmarking has not been previously adequately addressed in BH. Thus, the scope of the study was to apply and to calculate known indicators of reproductive performance and provide initial data as a reference for further application. The present study describes key reproductive performance indicators at the individual animal level in selected dairy farms of Una-Sana Canton.

The average numbers of heifers and dairy cows per visited farm is small in comparison with the average herd sizes in EU countries [16]. Considering limiting factors in the bovine industry in BH, especially small stocking density, maintaining a constant number of cattle per farm, and its gradual increase, is a difficult task for dairy farmers. Thus, the optimal replacement of the herds is a primary managerial effort for dairy farmers in BH. The rearing of young stock is the most common source of replacement to avoid the costs of purchasing new animals. Farmers sought to optimise the age at first insemination (AFI) to introduce their heifers in the (re)production as early as possible. However, the mean AFI was substantially different among selected dairy farms as shown in this study. This indicated the lack of planning the heifer management but also the lack of rearing standards and professional decision-making. Also, this finding highlights the need for continuous education of farmers in order to avoid the observed reproduction delays. In addition, there is a need for the introduction of heifer rearing goals and professional follow-up. Farms with a smaller number of heifers had a lower median AFI and AFC but also fewer heifers whose AFI and AFC deviates from the farm average. The observed trend indicatesthat this association may be partially explained by the farmer's adjusting the breeding programme to each heifer. On the other hand, farmers on farms with a greater number of dairy cows were directed toward more intensive milk production. It could mean that heifers were potentially bred at the predefined time, e.g., when reaching the certain body weight. However, individual variations of the AFI in such farms were not followed up, and more research is needed.

The age at first calving (AFC) in the selected dairy farms was partially influenced by the AFI (Figure 3). Achieving a lower AFC has been found to be associated with improved reproductive performance and lifetime production, as well as timely successive calvings [17]. Moreover, earlier studies have shown that the AFC may substantially contribute to the total rearing costs [18, 19]. We observed substantial differences in the AFC between visited farms and given the median AFC of 802 days; our findings suggest that some of the visited dairy farmers might be affected by some economic burden due to the elevated median AFC in relation with the general goal at 22 to 24 months (730 days) [20, 21].

This study reported an overall pregnancy proportion at first service of 40% although there was a wide interfarm variation. The pregnancy proportion at first service measured per farm (data not shown) coincided with the AFC, i.e., farms that experienced higher AFC also had lower pregnancy proportion. This trend is assumed to be related to farm management on smaller farms, as managers reported to follow the heifers more closely. Others have found that reproductive performance and health of dairy cattle is not universally better in smaller farms [22]. Furthermore, the cumulative pregnancy rate at the population level showed that one-third of all pregnancies happened after day-120 postpartum (Figure 4(b)). Although the median CCI of 101 days at the population level did not indicate the existence of reproductive problems in the selected dairy farms in Una-Sana Canton, the one-third of pregnancies happened after day-120, indicating animals with high CCI in each visited dairy farm. The median values of the CCI in such skewed distribution likely failed to demonstrate individual variations within the herd. On the other hand, the combination of these reproductive measures is of particular interest. It gives the possibility that animals which got pregnant after day-120 and cows with high CCI can be evaluated by veterinary services separately from the rest of the herd. Accordingly, the improvement of the veterinary advisory service and its structured approach to animals whose indicators suggest a reproductive problem arises as one of the primary goals in the dairy sector in BH. Although not commonly used in BH, timed AI can be introduced as an alternative cost-effective and viable solution for this group of animals, as previously reported [23, 24]. Animals with chronic reproductive problems could also be removed from the dairy herd although farmers reported that reproductive culling is not a common management practice in their farms in Una-Sana Canton.

The observed interservice interval of 18–24 days for more than two-thirds of repeat breeding heifers and multiparous animals at the population level indicated a very good oestrus detection. Remnant et al. (2018) reported that interservice interval of 19–26 days indicated that this period is the true latent distribution for the interservice interval with the optimal reproductive outcome, suggesting day-22 with the increased probability of conception [25]. However, we found that a total of 9.6% of interservice intervals were longer than 48 days (Figure 6). Apart from being farmer-dependent, the extension of interservice interval over the 48 days may be the result of some other reproductive problems, such as anoestrus [26]. In addition, such extension may be a result of the occurrence of embryonic or foetal death [27]. Our finding calls for targeted control of this group of repeat breeding cows. Importantly, caution is needed in the interpretation of our results. In the current study, however, interservice intervals were only calculated for farms that had satisfactorily written records, and thus, excluding some of the largest herds.

Artificial insemination (AI) was the dominant type of breeding for dairy heifers in the selected dairy farms. AI is exclusively performed by veterinarians or veterinary technical staff who visit the farms after the farmers' call. The timing of AI might be influenced by the farm demographics which farmers in this study reported to be of concern in relation to repeat breeding. In addition, most of the farmers reported that they have side income which resulted in inappropriate or less time for oestrus detection. Irrespective of satisfactory oestrus detection found in this study, this may in part have contributed to the persistence of repeat breeding animals. On the other side, the larger farms rely on nonfamily and seasonally employed labour. Considerable difference in farm workers' experience and competences could potentially influence the oestrus detection. Similarly, managing farm labour has been found to be of the greatest challenges in dairy farming [28]. Based on data from the farms visited in this study, managers of larger farms, i.e., farms with the greater number of animals, did not keep appropriate farm records in comparison with managers of small farms, and their ability to identify a reproductive problem at the individual animal level might be reduced. All visited farmers combined natural breeding and artificial insemination to reduce periods of repeat breeding. However, the results indicate that such managerial decision had either a slight or no effect on the CCI, i.e., the number of repeat breeders remained the same. Herds using natural services rarely register the number of services per pregnancy. However, in the current study, the mean NSP was 2.61, and this number reflects both natural services and artificial inseminations. This points to reproductive shortfalls compared with other studies [29, 30]. Farms with low pregnancy rates (first service and cumulative) at individual animal level had the highest NSP. Likely, the NSP was dependent on a large number of factors such as the oestrus display, oestrus detection, timing of service, sire fertility and sperm quality, subclinical diseases, and management features. Other studies are needed to investigate all aspects of increased NSP.

Farmers had different views on how long they should wait in restarting the cows' reproduction after calving, and consequently, we were not able to calculate the voluntary waiting period directly from the farm records. However, farmers reported that they usually wait for two consecutive oestruses, after which they restart with inseminations. This information is regarded imprecise since the period of voluntary waiting remains undefined in most cases. A recent study on management practices associated with reproductive performance in dairy cattle reported that the lack of a well-established VWP (<50 days) was associated with shorter CFI and consequently shorter CCI [31]. Other studies have advocated a voluntary waiting period of at least 60 days, and the timed AI has been advised to be 73 days [32]. Studies that are aimed at investigating and establishing the optimal VWP for dairy farms in BH are needed.

However, we were able to calculate the calving-to-first-service interval (CFI). The overall median CFI was 62.5 days and varied substantially among the selected dairy herds (Table 1). This is low compared to the report for Norwegian Red cattle which also shows a substantial variation in the population (85.3 days, SD ± 41.9) [33]. Further, within-farm variations substantially affected the estimation of the farm's median CFI in this study. The individual CFI can be extended by several factors such as nutrition [34, 35], endometritis [36], and poor oestrus detection. Elkjær et al. reported that even mild uterine infection could have an adverse effect on CFI [37]. Similarly, the uterine infection was also one of the observations associated with poor reproductive performance as presented in an earlier paper by our group [13].

The calving interval (CI) was calculated as the traditionally used fertility indicator, and the median CI of 385 days indicates a relatively good reproductive performance in selected dairy farms in Una-Sana Canton. Similar to our findings, another study reported an average CI of 12.6 months in the Norwegian Red cattle [33]. However, caution is needed in the interpretation of the CI. Since the CI is calculated retrospectively and represents the sum of all previous reproductive measures, it could be influenced by wide individual variations within the dairy herd. Given that our study revealed that one-third of pregnancies happened after 120 days in milking (Figure 4(b)) and the average gestation period, there is a legitimate expectation that CIs for such pregnancies were greater than 400 days. The identification of those animals is the primary aim of recording animal performance. Given that the system for animal identification and traceability has already been established in BH, our study indicates the need for upgrading such system with animal performance recording. A comprehensive recording system could help the veterinarians/advisors and farmers to determine if their reproductive management reflects the number of pregnant animals promptly. In addition to pregnancy recorded, the unavailable data of culled or sold individuals and 57% of individuals not included in the calculation due to the inappropriate written data might influence the actual CI of the investigated population. The calculated reproductive measures indicated an undulant trend in reproductive performance among selected dairy farms in Una-Sana Canton, which is similar to the recent breeding programme set by national authorities. This may be the optimal level of reproductive performance for this subset of farms, considering the study limitations.

### 4.1. External and Internal Validity of the Study

This study described key reproductive performance indicators in dairy farms in Una-Sana Canton. Given that dairy farmers have more or less the same managerial approach to dairy cattle rearing across the entire territory of the country, our findings may refer to other areas in BH. Also, the Simmental is the most common breed of cattle in BH, and thus, governmental and agricultural authorities have prescribed a breeding programme regarding maintaining and improving productive, reproductive, and exterior characteristics of this breed [38]. Reproductive goals in the breeding programme are set for AFI (14.5–16 months), AFC (24–26 months), average productive life (7-8 years), CCI (100 days), NSP (1.8), and CI (<376 days). Although results of this study show the undulant trend in reproductive performance in commercial dairy farms (Tables 1 and 2), there is a substantial similarity of reproductive performance in dairy cattle in Una-Sana Canton with reproductive goals set in the recent breeding programme. This is of great importance for future dairy operations in Una-Sana Canton; however, there are several aspects in reproduction that should be improved. Also, the obvious lack of records regarding reproduction and reproductive culling could influence the reproductive measure, and the actual reproductive performance could be weaker than shown in this study. In addition, the expected future intensification of the production in BH could, however, substantially contribute to the more serious decline in reproductive performance, as this trend is evident throughout the world [1]. The farms used in this study were farms with appropriate written records, and the external validity of this study is limited.

## 5. Conclusions

The recording of reproductive performance in dairy farms in Una-Sana Canton of BH is based on written or even memorising data, with no proper recording system in bigger farms. There is a need for an organised, regular, and more comprehensive recording system in the reproduction of dairy cattle among dairy farms in Una-Sana Canton and BH as a whole. Knowing the apparent reproductive indicators described in this study, the farmers and veterinary authorities in BH may identify and correct areas in their management that contribute to the reproductive underperformance in dairy cattle.

## Figures and Tables

**Table 1:** Reproductive measures and their median distribution in selected dairy farms in Una-Sana Canton.

Farm ID	CFI	CCI	CI
1	59.5^*∗*^(35 − 100)^*∗∗*^	110.5^*∗*^(39 − 223)^*∗∗*^	398^*∗*^(344 − 477)^*∗∗*^
2	62.5 (44–176)	96 (62–242)	350.5 (332–442)
3	50 (30–102)	94 (34–320)	388 (321–618)
4	62 (20–98)	131(50–342)	388 (335–473)
5	69.5 (39–389)	126 (44–389)	425.5 (328–606)
6	55 (38–130)	73 (38–215)	359 (324–491)
7	63 (43–209)	113 (52–235)	393.5 (339–445)
8	60 (41–93)	83 (42–134)	353.5 (322–435)
9	61 (42–121)	116 (59–329)	385 (337–537)
10	63 (44–145)	121 (67–235)	392 (346–497)
11	55.5 (21–84)	100.5 (21–185)	386 (341–444)
12	76.5 (38–277)	111.5 (38–470)	380 (344–584)
13	75 (52–106)	88 (42–138)	372 (335–765)
14	79 (60–134)	126 (60–239)	409 (350–560)
15	80 (25–142)	103.5 (56–188)	403 (334–444)
16	62.5 (60–276)	104 (61–387)	390.5 (340–769)
17	63 (53–124)	63 (19–207)	413 (343–483)

^*∗*^All intervals are given in days. ^*∗∗*^5^th^–95^th^ percentile range.

**Table 2:** Median annual distribution of reproductive parameters in the follow-up period 2009–2016 in selected dairy farms in Una-Sana Canton.

	2009	2010	2011	2012	2013	2014	2015	2016	Overall
*n*	6	17	25	39	72	97	125	75	234
CFI	62.5	63	62	61	63	60	62	66	62.5
*R*	42–86	36–408	40–104	31–121	40–121	38–106	38–143	40–169	31–408
*n*	7	17	24	39	72	95	127	a	234
CCI	63	101	98.5	99	102.5	103	118	a	101
*R*	42–158	36–506	46–218	43–255	54–221	39–244	52–326	a	36–506
*n*		7	15	24	38	67	87	109	234
CI	a	347	402	396	383	381	385	394	385
*R*	a	320–378	343–773	329–545	328–497	330–513	327–506	331–584	320–773

*R*, 5^th^–95^th^ percentile range; a, no data. All intervals (CFI, CCI, and CI) are given in days.

## Data Availability

The data used to support the findings of this study are available from the corresponding author upon request.

## Abbreviations

AI: artificial insemination
AFI: age at first insemination
AFC: age at first calving
NSP: number of services per pregnancy
ISI: interservice intervals
CFI: calving-to-first-insemination interval
CCI: calving-to-conception interval
CI: calving interval
